# Ultrasonic partial glossectomy

**DOI:** 10.1186/1758-3284-1-21

**Published:** 2009-06-24

**Authors:** Yoann Pons, Jérome Gauthier, Philippe Clément, Claude Conessa

**Affiliations:** 1 Head and Neck Surgery Department, Val of Grace's Academic Military Hospital, Paris, France

## Abstract

**Introduction:**

Partial glossectomy is the main treatment for tongue carcinoma. The resection of the tongue, which is a very vascularised tissue, requires a good hemostasis. The advantage of the harmonic scalpel is in combining sectioning and hemostasis in one single instrument, allowing a bloodless dissection of soft tissue. The aim of this prospective study was to evaluate the benefits and risks when using a harmonic scalpel in partial glossectomy.

**Subjects and Methods:**

In this prospective study conducted in a university hospital from march 2004 to Decemeber 2008, eighteen consecutive patients underwent a partial glossectomy with the use of harmonic scalpel. Results were compared with previous surgical procedures performed between September 2000 and February 2004 by monopolar hemostasis by our team (n = 12) when the harmonic scalpel was not available.

**Results:**

All 18 patients underwent partial glossectomy with the harmonic scalpel as the only instrument of section and hemostasis. The median blood loss was of 0 mL. The median operative time was 29 minutes (16 minutes less than partial glossectomies performed with conventional hemostasis. P < .001). No operative complications occurred. Two post-operative bleedings (5 days and 7 days after the glossectomy) occurred necessitating a new surgery to ligate the lingual artery. The margins of the resection were acceptable and no recurrence appeared.

**Conclusion:**

The harmonic scalpel makes it fast and easy to perform a partial glossectomy with no bleeding. Ligation of the lingual artery (when it is visualized during the dissection) should be performed because of the frequency (more than 10% in our series) and because of the potential gravity of a lingual post-operative bleeding.

## Introduction

Parial glossectomy is the main treatment for tongue carcinoma. The resection of the tongue, which is a very vascularised tissue, requires a good hemostasis. Since its development in the late 1960s, ultrasonic dissection has been used in a growing number of surgical procedures. Initially it was developed for laparoscopic surgery [1,2]. Head and neck surgeons then used it for thyroid surgery [3,4], in which the technology was recognized as being particularly quick and efficient, before using it in a number of other head and neck surgical procedures [5,6].

The advantage of the harmonic scalpel is in combining sectioning and hemostasis in one single instrument, allowing a bloodless dissection of soft tissue (vessels, muscle, fat tissue). The ultrasonic scalpel is made up of three parts: a generator, a hand piece and the scissors. It employs an ultrasonic frequency to generate mechanical energy as a result of vibrations (at a frequency of 55500 Hz), which breaks down the hydrogen bonds in tissue protein resulting in a sticky coagulum that seals off blood vessels.

This technology for partial glossectomy has been described somewhat in the literature. In 2000, Sherman et al reported on the first partial glossectomy carried out by ultrasonic scissors.^5 ^A letter to the editor was written on the subject by To et al in 2001 [7]. Since then, two series have been reported on: one with 25 patients in 2005 by Metternich et al [8], and one with 13 patients in 2005 by Yuen at al [9].

The aim of this prospective study was to evaluate the benefits and risks when using a harmonic scalpel in partial glossectomy.

## Subjects and methods

### Patients

In this prospective study, all patients underwent partial glossectomy by harmonic scalpel between March 2004 and December 2008. In total, 18 consecutive partial glossectomies were carried out during this time. There were 15 men and 3 women with a mean age of 61 (range, 40–82) all of who had squamous cell carcinoma of the tongue. The distribution of the tumours was as follows: T1N0M0 (n = 10), T2N0M0 (n = 4), T2N1M0 (n = 2) and T2N2aMO (n = 2). None of them underwent preoperative chemotherapy or radiation, and four of them underwent a postoperative radiotherapy. The mean tumour diameter (estimated by MRI for each tumour) was 21 mm (10–30 mm). In 10 cases the tumour was situated only on the mobile part of the tongue; in 3 cases it was situated only on the base tongue; and in 5 cases the tumour was at the junction mobile tongue-base tongue.

### Surgical procedure

All the operations were carried out, or supervised, by the last author. All the patients underwent a partial glossectomy performed by the harmonic scalpel (Harmonic ACE 23P). The patients were operated on by transoral approach (n = 15) or cervical approach for the tumour situated on the base of the tongue (n = 3).

### Methods

The operative data collected were: the duration of the tongue resection, the quantity of bleeding by the succion and the operative incidents. The postoperative data collected were: the pain (measured by visual analogical scale and the analgesics consummation), the margins of tumor removal, the necrosis layer due to the cauterization of the harmonic scalpel at the level of the cut, and the postoperative complications. All the patients signed a consent form. Their confidentiality was protected. The Val of Grace's institutional review board approved this study.

The mean operative time for partial glossectomies in this series was compared to the mean operative time for the partial glossectomies that we performed between 2000 and 2004 using monopolar hemostasis (n = 12) when the harmonic scalpel was not available. These durations were compared using a Student t-test.

## Results

The mean operative time for ultrasonic partial glossectomy was 29 ± 12 minutes, showing a decrease in time of 16 minutes from the report of the partial glossectomy with monopolar hemostasis (p < .001) (Table 1). This duration measured only the necessary time for the resection of the tongue tumor. It did not include the necessary time for the approach and the reconstruction. The median blood loss for the partial glossectomy during intervention was 0 mL and no operative bleeding occurred.

**Table 1:** Collected data (mean and standard deviations).

**results**	**Harmonic scalpel**	**Monopolar resection**
**Operative time (min)**	29 ± 12	35 ± 12
**Résection layer (mm)**	16 ± 3	
**Necrosis layer (mm)**	8 ± 3	
**Diet begining (days)**	5 ± 1	
**Normal diet (days)**	8 ± 1	

All of the tumor resections were performed in sano. The mean security margin was 16 ± 3 mm. The mean necrosis layer due to the cauterization of the harmonic scalpel at the level of the cut was 8 ± 3 mm.

Postoperative pain was minimal in all cases except one. This one patient was a drug addict who needed to be administrated morphine for 5 days after the operation to control the pain. All other patients benefited from simple analgesics and did not receive morphine.

Two postoperative bleedings occurred: one on the fifth day and the other on the seventh day after the partial glossectomy. In both cases, a new surgery was necessary to ligate the lingual artery. In one case, general anaesthesia with intubation was possible. In the other case, the amount of bleeding prevented us, because of the risk of inhaling blood at the moment of intubation, from carrying out the haemostasis by three X points without anaesthesia.

The weak postoperative pain allowed patients to drink the day after the operation, to eat cold or warm foods after 5 days and to return to a normal diet after 8 days. This increase in diet was carried out under the control of a speech therapist when the lingual resection was extended to the base tongue, which made swallowing difficult.

## Discussion

**Figure 1:**
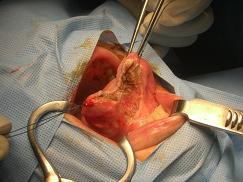
**Operative view**. Bloodless ultrasonic removal of a carcinoma of the mobile tongue.

The tongue is a very vascularised tissue. Nevertheless, the ultrasonic partial glossectomy was a fast, easy and bloodless procedure. However, this lingual resection technique should be performed on small tissue layers (around 5 mm) in order to achieve the most efficient outcome possible and to insure an efficient hemostasis of the lingual vessels (Figure 1). This procedure allowed a fine removal of the tongue tumour.

In our series, no operative bleeding occurred, though a few cases of bleeding have been recorded in the medical literature [8,9]. The reported cases were easily controlled. Therefore, even if the quality of the harmonic hemostasis had been shown, as for the blood vessels of less that 3 mm in diameter (which is the case for the lingual vessels, even at the base of the tongue), with a pressure inferior to 226 mmHg [10], this dissection can be complicated by bleedings requiring vessels ligature and/or additional monopolar or bipolar hemostasis.

Late postoperative bleeding was relatively frequent in our series (2 cases out of 18 partial glossectomies) that had not been previously found in literature. They all happened after the fifth postoperative day, at the time when the scar fell. These lingual bleedings, stemming from the lingual artery, were severe and they made it difficult to intubate the patient without the risk of blood inhalation at the time of the anaesthetic induction. For this reason, we had to carry out hemostasis without anaesthetic in 1 of the 2 cases. In both cases the bleeding arose on the cut of the tongue. The section-cauterization of the lingual artery was performed in a muscular, elastic, contractile and mobile structure that can put tension on the cut zone and therefore weaken it. Moreover, this cut zone is superficial, and it can come in contact with saliva and food, thus weakening the coagulum. These incidents of late postoperative lingual bleedings, like the palatine tonsillectomy bleedings incidents, are a classic complication at the moment when the scar fell. Thus, we recommend to ligate the lingual artery when it can be visualized during the tongue dissection and we encourage the hospitalization of patients for at least seven days after the partial glossectomy.

The ultrasonic partial glossectomy provoked only little pain and patients took simple analgesics (no morphine except in one case) to control the pain. The use of the harmonic scalpel is known to give little postoperative pain, much less than the hemostasis carried out with the help of monopolar or bipolar cauterization [11-13]. This reduced pain is due to the fact that ultrasonic hemostasis is carried out at a low temperature between 50 – 100°C compared to 150 – 400°C for the monopolar and bipolar electrocoagulation [12]. It thus results in a slight thermal diffusion of adjacent structures. This hemostasis is performed at a low energy and temperature leading to less postoperative pain and faster healing [11-14].

**Figure 2:**
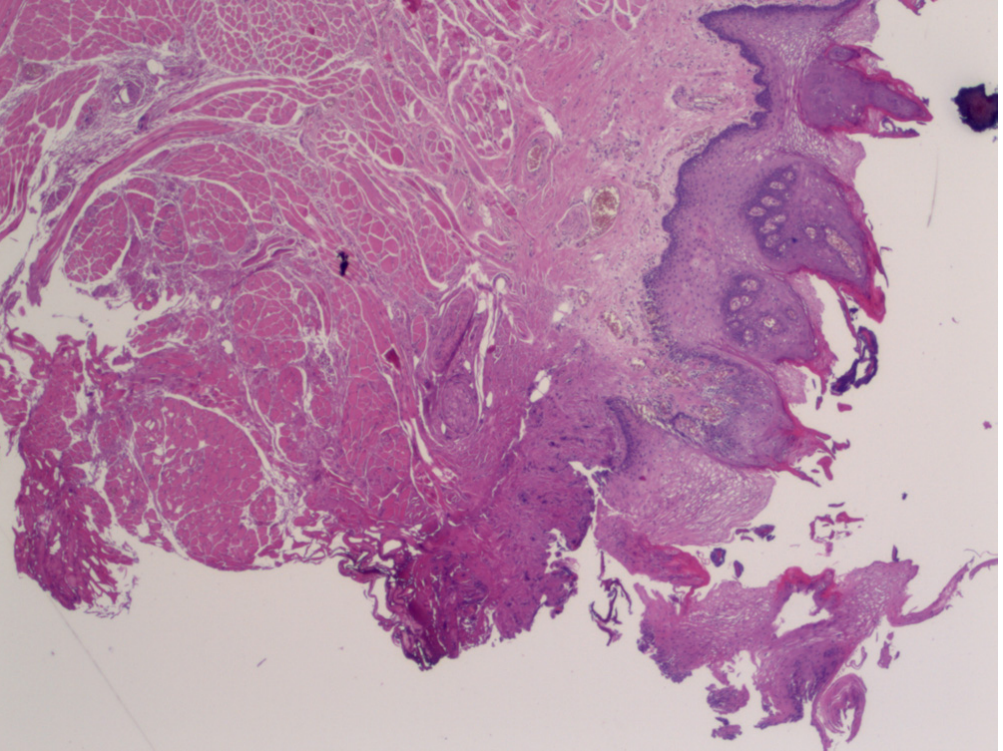
**Microscopic view (× 25) of the necrotic layer at the level of the cut of the tongue (at the bottom on this picture)**. This layer (about 0.5 mm here) cannot be interpreted by the pathologist.

Because the tongue dissection was performed without bleeding, all the tumour removals have been carried out with an acceptable margin resection (which mean was 16 mm) and no recurrence appeared. The pathologist can hardly interpret the cut zone because of the necrosis induced by the hemostasis. The use of the harmonic scalpel reduced this zone to 0.8 mm on average (Figure 2). But this section did not prevent the surgeon from performing the re-cut on the border of the tumor. This measurement is in accordance with the data previously reported in the literature (0 to 2 mm). It is a very weak layer, especially compared to the layer burnt by the monopolar and the bipolar cauterization which provokes, at the level of the cut, a layer of necrosis of 6 to 8 mm [10]. Thus, the use of the harmonic scalpel facilitated the job of the pathologist in interpreting the margins of the resection.

The overall cost of the consumable materials (essentially represented by the price of a pair of harmonic scissors) was largely compensated for by the reduction of the operative time which leads to a reduction of costs for the rental of the surgical box and the surgical team [9]. The price for the generator was not counted in this study because it is a piece of technology used for several specialties in our hospital (visceral surgery, urology) and thus its price would be calculated in a large number of surgical interventions.

## Conclusion

The harmonic scalpel makes it fast and easy to perform a partial glossectomy with no bleeding. The ligation of the lingual artery (when visible during the dissection) should be done because of the great frequency (more than 10% in our series) and the potential gravity of late postoperative bleeding of the lingual artery.

## Consent

The Val of Grace's Review Board approved this study.

## Competing interests

The authors declare that they have no competing interests.

## Authors' contributions

YP was the main redactor of the manuscript. JG helped him to redact the manuscript. PC revised first the manuscript. CC defined the study design and performed the final revision of the manuscript. All authors read and approved the final manuscript.
